# Assaying protein palmitoylation in plants

**DOI:** 10.1186/1746-4811-4-2

**Published:** 2008-01-11

**Authors:** Piers A Hemsley, Laura Taylor, Claire S Grierson

**Affiliations:** 1 School of Biological Sciences, University of Bristol, Bristol, UK

## Abstract

**Background:**

Protein S-acylation (also known as palmitoylation) is the reversible post-translational addition of acyl lipids to cysteine residues in proteins through a thioester bond. It allows strong association with membranes. Whilst prediction methods for S-acylation exist, prediction is imperfect. Existing protocols for demonstrating the S-acylation of plant proteins are either laborious and time consuming or expensive.

**Results:**

We describe a biotin switch method for assaying the S-acylation of plant proteins. We demonstrate the technique by showing that the heterotrimeric G protein subunit AGG2 is S-acylated as predicted by mutagenesis experiments. We also show that a proportion of the Arabidopsis alpha-tubulin subunit pool is S-acylated *in planta*. This may account for the observed membrane association of plant microtubules. As alpha-tubulins are ubiquitously expressed they can potentially be used as a positive control for the S-acylation assay regardless of the cell type under study.

**Conclusion:**

We provide a robust biotin switch protocol that allows the rapid assay of protein S-acylation state in plants, using standard laboratory techniques and without the need for expensive or specialised equipment. We propose alpha-tubulin as a useful positive control for the protocol.

## Background

S-acylation is a reversible post-translational modification of cysteine residues in proteins. It is also known as protein palmitoylation, but this term is less accurate because a variety of acyl lipid groups (e.g. palmitoyl, stearoyl) can be added to cysteine residues. S-acylation is sometimes confused with acetylation, which is the addition of acetyl groups to amines, but the two are fundamentally different processes. S-acyl groups associate with membranes and are frequently found on proteins associated with membrane microdomains [1,2]. There have been three positive identifications of S-acylated plant proteins. OsCPK2, a protein kinase from rice, was labelled by H^3^-palmitic acid *in vivo *[3]. ROP10, a type II small GTPase, was modified by H^3^-palmitoyl-CoA *in vitro *[4] and ROP6, a type I small GTPase, was shown to be primarily modified by stearic acid by gas chromatography [5]. Other proteins such as RIN4 [6], AGG2 [7,8] and various calcium-dependent [9] and calcium-sensing kinases [10] have been predicted to be S-acylated based on mutational studies (replacing relevant cysteine residues), tests for membrane association, and the use of S-acylation inhibitors, but no direct evidence exists for their S-acylation.

Computational methods for predicting S-acylation sites have been developed such as Terminator2 [11,12] which attempts to identify S-acylation sites within the first 20 amino acids of a protein and CSS-Palm [13] and NBA-Palm [14] which aim to identify S-acylation sites throughout a protein. As yet no biochemical validation of previously unknown S-acylated proteins being identified using these programs has been reported in the literature.

Once potential S-acylation sites have been identified, either through predictive or experimental means, a method for verification of the site is required. A novel method for identifying S-acylation sites in mammals was developed [15], termed the biotin switch protocol, which allowed accurate and specific assaying of S-acylation sites but did not work reliably in all situations. Adaptations and revisions of this method have since been developed and applied to budding yeast [16,17] and plants (this study) and are reliable and work well with all proteins, without further optimisation. The method described here is equally applicable for the detection of S-acylation of native or transgenic proteins from plant tissue. This is an advance on previous approaches which either require large amounts of starting material, expensive chromatographic and mass spectroscopy equipment [5] or incubation of cell cultures or heterologously expressed proteins in the presence of H^3^-palmitic acid followed by immunoprecipitation and autoradiography [18]. This latter method is expensive, hazardous and extremely time consuming, typically requiring upward of 6 weeks for exposure of the autoradiograph.

Here we present modifications and refinements of the biotin switch method [15] and a detailed protocol to allow rapid and affordable identification of S-acylation sites in plant proteins. This protocol represents an improvement over the standard assays for S-acylation as it does not involve the use of radioactive chemicals, addition of exogenous chemicals for labelling, or pharmacological treatments commonly used to aid in label incorporation. We have used the biotin switch assay to show that the α-subunits of Arabidopsis tubulin are S-acylated. As α-tubulin is ubiquitously expressed, and anti-α-tubulin antibodies are readily available, it can be used as an internal control during biotin switch acylation assays without any extra processing of protein samples. We have also assayed the S-acylation state of the heterotrimeric G-protein γ 2 subunit AGG2. It has been suggested that AGG2 might be S-acylated, because AGG2 mutants lacking a cysteine residue in the C terminus are incorrectly localised in plant cells [7,8].

## Results

**Figure 1:**
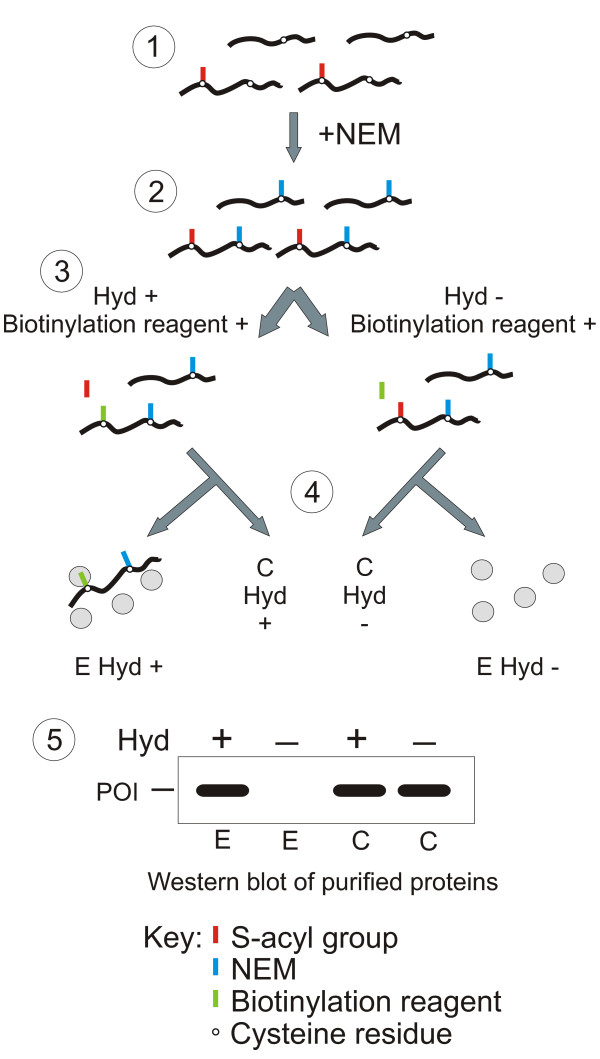
**Simplified overview of the biotin switch assay for detecting S-acylated proteins**. For simplicity only four proteins are shown in this scheme, two S-acylated, and two not. In reality all of the proteins from a lysate are subjected to this treatment resulting in the purification of all S-acylated proteins in a sample. (1) Cell lysates containing proteins (black lines) with cysteine residues (open circles) may have acyl groups (red lines) attached through a thioester bond (S-acylation). (2) Lysates are treated with the sulfhydryl reactive reagent N-ethylmaleimide (NEM, blue line) to block free cysteines. (3) The sample is then divided into 2 equal portions and one treated with the S-acyl group cleavage reagent hydroxylamine (Hyd+) and one without (Hyd-). Both samples are then treated with sulfhydryl reactive biotin (green line). Free sulfhydryls liberated by hydroxylamine treatment (Hyd+) are labelled with sulfhydryl reactive biotin to form a biotinylated cysteine residue. Samples incubated in the absence of hydroxylamine (Hyd-) do not undergo biotinylation as free sulfhydryls are not generated. (4) Following biotinylation a sample of each reaction is removed to act as a column loading control (C Hyd+ and C Hyd-). The remaining sample is loaded onto neutravidin beads and biotinylated proteins, representing S-acylated proteins in the lysate from step 1, are purified (E Hyd+ and E Hyd-). (5) After elution from the neutravidin beads protein samples are separated by SDS-PAGE and the protein of interest (POI) can be assayed for S-acylation by western blot using an anti-POI antibody. The pattern of signals expected of an S-acylated protein assayed by this method is shown. Proteins that were not S-acylated would produce a signal from C Hyd+ and C Hyd- samples but not from E Hyd+ and E Hyd- samples. Proteins that were biotinylated *in planta*, rather than as a result of the biotin switch protocol, would produce a signal in both the E Hyd+ and E Hyd- samples. In these cases the level of *in planta *biotinylation will be evident from the E Hyd- lane.

The biotin switch method, summarised in Figure 1 and fully described in the methods, can be applied to any protein suspected of being S-acylated. Essentially the protocol involves the substitution of acyl groups with biotin, affinity purification of biotinylated proteins, followed by western blotting to determine the level of acylation of the protein in question. A model western blot is illustrated at the bottom of Figure 1. The blot requires an antibody directed against one of the following: the protein of interest; or an epitope tag such as FLAG, MYC, or 6xHIS; or a fluorescent protein variant in the case of fluorescent protein fusions.

### Verification of the biotin switch protocol using TIP1

We previously demonstrated autoacylation of the Arabidopsis S-acyltransferase TIP1 using an H^3^-palmitic acid protocol [18]. We tested whether TIP1 autoacylation could also be detected by the new biotin switch protocol. In the H^3^-palmitic acid assay, TIP1 was heterologously expressed in yeast, and covalently bound exogenously supplied H^3^-palmitic acid. The TIP1 C^401^A point mutant did not bind H^3^-palmitic acid, suggesting that auto-acylation required cysteine 401 [18].

**Figure 2:**
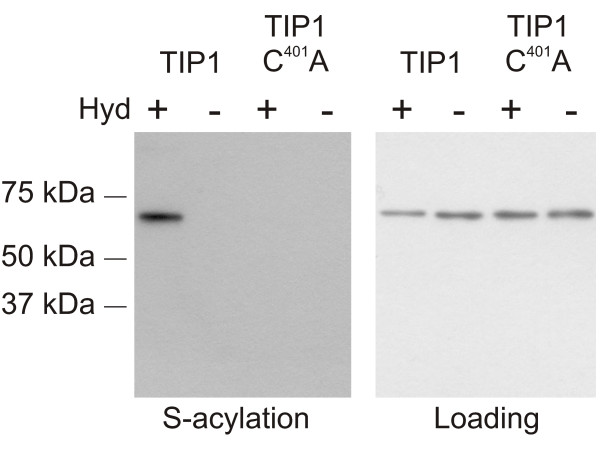
**The biotin switch protocol detects C^401^A-dependent S-acylation of TIP1**. ysates of yeast expressing TIP1, or the point mutant TIP1 C^401^A, were put through the biotin switch protocol summarised in Figure 1. Samples were treated with (Hyd+) or without (Hyd-) the thioester cleavage reagent hydroxylamine. The loading controls show that equal amounts of TIP1 and TIP1 C^401^A were loaded onto neutravidin beads. The lanes labelled 'S-acylation' show the levels of TIP1 and TIP1 C^401^A recovered from the neutravidin beads, and therefore originally S-acylated. TIP1 and TIP1 C^401^A have masses of 70 kDa. The results show that Arabidopsis TIP1 binds acyl groups by a covalent thioester bond but TIP1 C^401^A does not, consistent with previous results using a H^3^-palmitic acid S-acylation assay [18].

We tested the biotin switch assay using exactly the same yeast strain expressing epitope tagged TIP1 and TIP1 C^401^A that we had used for the H^3^-palmitic acid protocol [18]. The biotin switch assay uses N-ethylmaleimide (NEM) to block free sulfhydryls followed by cleavage of thioesters with hydroxylamine and labelling of liberated sulfhydryls with biotin (Figure 1). In the negative controls sulfhydryl reactive biotin is still present but Tris, which does not cleave thioester bonds, replaces hydroxylamine. The method allows the levels of hydroxylamine labile thioester bonds, indicative of S-acylation, to be assayed [15]. The biotin switch protocol was performed as described in the methods section and was able to demonstrate that TIP1, but not TIP1 C^401^A, is S-acylated (Figure 2) as we previously reported [18]. TIP1 C^401^A was not able to bind acyl groups and, despite containing 17 other cysteine residues, signal was not detected from TIP1 C^401^A samples by this protocol. This demonstrates that the hydroxylamine treatment used here is specific for thioester modified cysteines. No signal is detected in the absence of hydroxylamine, indicating the full blocking of non-S-acylated cysteine residues [15]. Thus we conclude that in our hands the biotin switch protocol is able to specifically detect the S-acylation of protein cysteine residues in a manner consistent with previous data [18].

### A fraction of α-tubulin is associated with membranes

**Figure 3:**
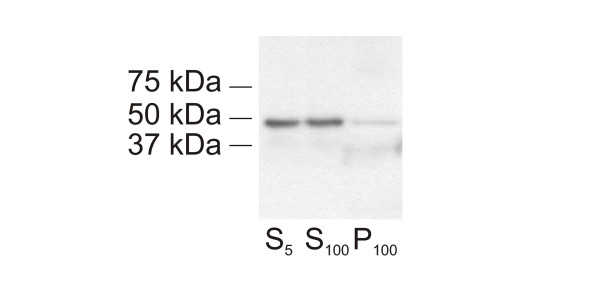
**A fraction of Arabidopsis α-tubulin is associated with membranes**. Membrane preparations from Arabidopsis roots indicate that a fraction of α-tubulin is associated with membranes. S5 = soluble fraction after centrifugation at 5,000 × g, S100 = soluble fraction after centrifugation at 100,000 × g, P100 = insoluble (membrane) fraction after centrifugation at 100,000 × g. α-tubulin has a mass of approximately 49 kDa.

Tobacco [19], Arabidopsis [20] and Cauliflower [21] α-tubulin has been shown to be associated with membrane fractions of cells and to be resistant to high salt treatments and non-ionic detergent treatments that remove peripheral and membrane associated proteins [19,21]. To verify that this applies to Arabidopsis microtubules we prepared soluble and insoluble fractions of Arabidopsis cell lysates after centrifugation at 100,000 × g. Western blotting indicated that a portion of Arabidopsis α-tubulin was membrane associated (Figure 3) in agreement with earlier work [20].

### Arabidopsis α-tubulin is S-acylated

**Figure 4:**
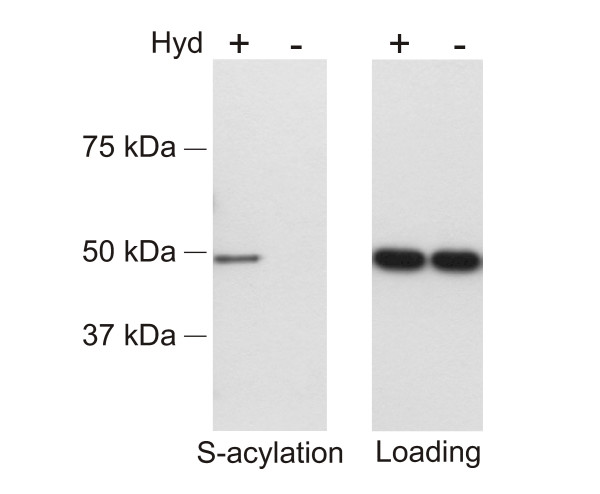
**Arabidopsis α-tubulin is S-acylated**. Subjecting Arabidopsis root whole cell lysates to the biotin switch protocol demonstrated that α-tubulin is S-acylated. Samples were treated with (Hyd+) or without (Hyd-) the thioester cleavage reagent hydroxylamine. The loading controls show that equal amounts of α-tubulin were loaded onto neutravidin beads. The lanes labelled 'S-acylation' show the levels of α-tubulin recovered from the neutravidin beads, and therefore originally S-acylated. α-tubulin has a mass of approximately 49 kDa. The results indicate that α-tubulin is S-acylated *in planta*.

A subset of α-tubulin subunits from rat brain lysates have been shown to be S-acylated (using excess H^3^-palmitoyl-CoA) and the majority of S-acylated tubulin is membrane associated [22]. We tested the S-acylation state of native α-tubulin from Arabidopsis roots as described in the methods. Our data (Figure 4) demonstrated that Arabidopsis α-tubulin was S-acylated.

### Arabidopsis AGG2 is S-acylated

**Figure 5:**
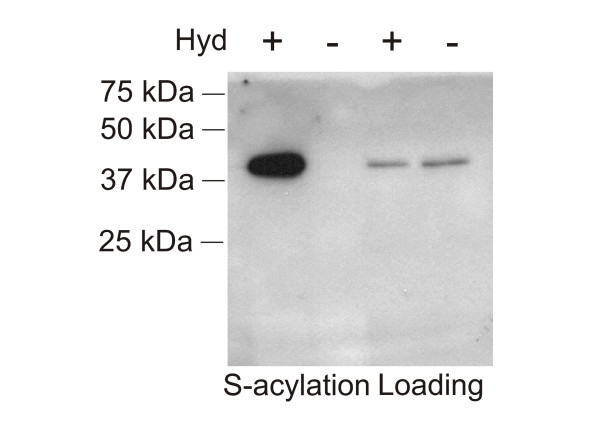
**Arabidopsis AGG2 is S-acylated**. Total membranes were purified from Arabidopsis plants expressing a FLAG-YFP-AGG2 fusion from the 35S promoter and subjected to the biotin switch assay. Samples were treated with (Hyd+) or without (Hyd-) the thioester cleavage reagent hydroxylamine. The loading controls show that equal amounts of AGG2 were loaded onto neutravidin beads. The lanes labelled 'S-acylation' show the levels of AGG2 recovered from neutravidin beads, and therefore originally S-acylated. FLAG-YFP-AGG2 has a mass of approximately 40 kDa. The results indicate that AGG2 is S-acylated *in planta*.

Arabidopsis AGG2 has been predicted to be S-acylated [7,8]. We have shown, using the approach described in the methods, that epitope-tagged AGG2 expressed from the 35S promoter in WT Columbia-4 plants was indeed S-acylated (Figure 5).

## Discussion

Here we demonstrate a successful biotin switch assay for the S-acylation of plant proteins, and use it to show that α-tubulin and AGG2 are S-acylated *in planta*. Plant microtubules are known to associate with membranes and to be resistant to treatments that would remove peripherally associated membrane proteins or disrupt protein-protein interactions [19-21]. It will be interesting to examine where, when and how α-tubulin is S-acylated and how this impacts upon microtubule function and dynamics. This is also the first demonstration of S-acylation of α-tubulin in any *in-vivo *system that does not rely upon the addition of exogenous label, or the use of pharmacological treatments to aid in label incorporation, to determine S-acylation of α-tubulin. Oryzalin treatment of live cells removes tubulin from the membrane but treatment of purified membranes with oryzalin has no effect on membrane-bound tubulin [19], suggesting that a soluble factor is required to remove tubulin from the membrane. These results are consistent with the idea that the membrane-association of tubulin is due to S-acylation, as the enzymes required to remove the S-acyl groups, palmitoyl protein thioesterases, are soluble [23].

AGG2, part of an Arabidopsis heterotrimeric G-protein, is predicted to be S-acylated based on mutagenesis data [8]. Here we have shown that AGG2 is indeed S-acylated confirming the usefulness of the biotin switch method.

A very small number of proteins are biotinylated *in planta *[24] and will be selected during affinity purification, so care should be taken if the protein of interest is suspected of being both S-acylated and biotinylated *in vivo*. In these cases the level of *in planta *biotinylation will be evident from the negative control (Figure 1).

There are many potential variations on the protocol presented here. Different biotinylation reagents are available such as Maleimide PEO_11 _biotin (Pierce). Maleimide PEO_11 _biotin, while not cleavable like Biotin-HPDP (which allows for mild elution from neutravidin beads), has a long spacer arm, is water soluble and can increase the solubility of modified proteins (manufacturer's data). This may make it more useful for the analysis of highly hydrophobic or bulky proteins where stearic hindrance may prevent neutravidin binding or the protein in question is difficult to solubilise. A modification of this protocol has also been developed for proteomics to identify all of the S-acylated proteins from yeast [25] and plants (Hemsley, Weimar, Dupree and Grierson, unpublished).

## Conclusion

Here we provide an adaptation of the biotin switch protocol applicable to plants. We have used it to confirm AGG2 S-acylation and show for the first time that Arabidopsis α-tubulin is S-acylated *in planta*. S-acylation of α-tubulin can be used as an internal control for the analysis of the S-acylation state of other proteins. No further processing of samples is required as the protocol purifies all S-acylated proteins and then the specific proteins of interest are identified by western blot. This means that the S-acylation state of the protein of interest and of α-tubulin can be determined simultaneously. The use of α-tubulin also allows any laboratory to get the protocol working in a reproducible fashion before embarking on important experiments as no special plant material is required.

## Methods

### General protocol for the biotin switch assay using plant material

A detailed protocol suitable for use in the lab is provided in Additional file 1. Briefly, plant tissue from plants grown as previously described [26] was ground to a fine powder in liquid nitrogen and resuspended in 500 μl lysis buffer. Samples were incubated for 1 hour at 4°C followed by centrifugation at 4°C, 500 × g to remove insoluble material. One mg of protein was incubated overnight with 25 mM NEM at 4°C to reduce proteolysis while allowing free sulhydryls to be blocked. Proteins were precipitated at room temperature using methanol/chloroform [27]. The pellet was resuspended in 200 μl resuspension buffer and the solution divided into two equal aliquots. One aliquot was combined with 800 μl of 1 M fresh hydroxylamine, 1 mM EDTA, protease inhibitors and 100 μl 4 mM biotin-HPDP (Thermo Scientific). As a control the remaining aliquot was treated identically but hydroxylamine was replaced with 50 mM Tris pH 7.4. Proteins were precipitated and resuspended in 100 μl of resuspension buffer. 900 μl PBS containing 0.2% Triton ×-100 was added to each sample, aliquots were removed as a loading control, and the remaining reactions were incubated with 15 μl of high capacity neutravidin-agarose beads (Thermo scientific). The neutravidin beads were washed twice with 1 ml PBS containing 0.5 M NaCl and 0.1% SDS and once with 1 ml PBS. Proteins were eluted by heating at 95°C in 25 μl 2× SDS sample buffer containing 1% 2-mercaptoethanol v/v. Samples were analyzed by SDS/PAGE and Western blotting on PVDF membranes as described below.

### TIP1 biotin switch assay for S-acylation in yeast

Yeast cells expressing FLAG epitope tagged TIP1 were grown in 50 ml selective galactose medium to O.D._600 _of 0.8 as described previously [18] and harvested by centrifugation (3,000 × g, 4°C for 15 minutes). Yeast cells were disrupted by glass bead lysis in 500 μl lysis buffer without Triton ×-100. Following lysis Triton ×-100 was added to 1% and incubated at 4°C for 1 hour. The lysate was centrifuged for 10 minutes at 500 × g to remove cellular debris and the supernatant retained. Protein concentration was assayed and the remainder of the biotin switch assay followed as described above. Membranes were blocked in TBS containing 0.05% Tween-20 (TBS-T, Sigma) and 5% non-fat milk powder (TBS-T 5% milk) and probed with 1:1,000 dilution of M2 anti-FLAG HRP conjugate (Sigma) in TBS-T 3% milk. The membrane was washed 3 times in TBS-T and 2 times in TBS for 5 minutes each. Membranes were exposed to film after incubation with ECL reagents according to the manufacturer's instructions (Amersham).

### Assay for α-tubulin membrane-association

500 mg root tissue was harvested from 10 day old plants, snap frozen in liquid nitrogen, ground to a fine powder and resuspended in 5 ml TBS with protease inhibitors (Calbiochem) and 1 mM EDTA and briefly vortexed. After filtration through miracloth samples were centrifuged at 5,000 × g for 10 minutes at 4°C. The supernatant was retained and centrifuged at 100,000 × g for 1 hour at 4°C. The supernatants were removed, pooled and combined with 1/10 volume 10% SDS while the pellet was resuspended and washed in 0.1 M Na_2_CO_3_. Pellet fractions were combined and recentrifuged and washed once with TBS before being resuspended in TBS containing 1% SDS. Protein concentration was determined using the BioRad DC method. 100 μg of each fraction were separated by SDS-PAGE and blotted for α-tubulin as described below.

### Western blotting for α-tubulin

Membranes were blocked in TBS-T 5% milk for 1 hour at room temperature. α-tubulin was detected using the TU-01 antibody (AbCam, Ab7750) [20] in TBS-T 3% milk at a 1:1,000 dilution for 1 hour at RT followed by 3 washes with TBS-T. Anti-mouse HRP conjugate (Zymed Research) was diluted 1:2,000 in TBS-T 3% milk and incubated for 45 minutes at RT. The membrane was washed 3 times in TBS-T and 2 times in TBS for 5 minutes each. Membranes were exposed to film after incubation with ECL reagents according to the manufacturer's instructions (Amersham).

### S-acylation of AGG2

The AGG2 cDNA coding region was fused in-frame to the c-terminus of YFP in pENTR FLAG YFP and recombined into pCAMBIA 1300 35S EC (Gift of Dr. C. Lazarus, University of Bristol) using the Gateway system (Invitrogen). Col-4 plants were transformed by floral dip [28] and selected as described [29]. Total membranes were prepared from leaf tissue of transgenic plants. 2 g of fresh leaf tissue was ground in liquid N_2 _to a fine powder, mixed with 5 ml of PBS with protease inhibitors (Calbiochem), 25 mM NEM and 1 mM EDTA and briefly vortexed. After filtration through miracloth samples were centrifuged at 5,000 × g for 10 minutes at 4°C. The supernatant was further centrifuged at 100,000 × g for 1 hour at 4°C. Pellets were briefly washed with PBS before being resuspended in PBS containing 1% Triton ×-100, protease inhibitors, 1 mM EDTA and 25 mM NEM. Protein concentration was determined with the BioRad DC kit and 1 mg of protein was used in the S-acylation assay describe above for plant tissue. Western blotting was performed using anti-FLAG HRP as described above for the TIP1 S-acylation assay.

## Competing interests

The author(s) declare that they have no competing interests.

## Authors' contributions

PAH developed and adapted the biotin switch assay for efficient and routine use with plant material, performed all experimental procedures and wrote the manuscript. LT transformed, selected and maintained AGG2 expressing plants. CSG coordinated the project and co-wrote the manuscript. All authors have read and approved the final text.

## Supplementary Material

Additional file 1 General lab protocol for the biotin switch assay. A detailed version of the biotin switch assay protocol for use in the laboratory. Click here for file
