# Supplementary material for: Assaying protein palmitoylation in plants
Source: Plant Methods. 2008 Jan 11;4:2. doi: 10.1186/1746-4811-4-2 (PMC2244627; doi:10.1186/1746-4811-4-2)
Supplement: Additional file 1 — General lab protocol for the biotin switch assay. A detailed version of the biotin switch assay protocol for use in the laboratory. [file 1746-4811-4-2-S1.doc]

**General Lab protocol for the biotin switch assay.**

The quantity of tissue used depends upon the level of expression of the target protein and the expected level of S-acylation. We recommend 250-500 mg of plant tissue for proteins expressed at high levels. If membrane purification to enrich S-acylated proteins is required for analysis then we suggest using 2 g of plant tissue. In the case of assaying for α−tubulin 500 mg of root tissue from plate grown plants was used. If the level of acylation of a protein is expected to be low then the protocol can be scaled up to allow more protein to be used initially (e.g. 2 mg starting protein).

1. Grind snap frozen plant tissue in liquid nitrogen to a fine powder and resuspend in 500 µl lysis buffer (N-ethylmaleimide prepared fresh before each use).
2. Incubate for 1 hour at 4 °C on a roller table.
3. Centrifuge at 4 °C, 500 x g for 10 minutes to remove insoluble material.
4. Determine protein concentration using a protein concentration assay kit compatible with 1% Triton X -100.
5. Combine one milligram of protein with lysis buffer to a total volume of 1ml and incubate overnight at 4 °C on a roller table. In some cases addition of Saponin to 0.5 % can increase blocking efficiency and S-acylated protein extraction.
6. Precipitate proteins at room temperature using methanol/chloroform [26] by adding 3 vol methanol, 1 vol chloroform and 4 vol water, mixing thoroughly and centrifuge at 10,000 x g for 30 minutes at 14 °C.
7. Remove and discard the upper phase without disturbing the interface and add 4 volumes of methanol, mix and incubate at -20°C for 20 minutes.
8. Centrifuge for 20 minutes at 5,000 x g at 4 °C.
9. Pour off the supernatant and air-dry the pellet for 10 minutes at room temperature.
10. Resuspend the pellet in 200 μl of resuspension buffer by sonication in a sonicating water bath for 10 minutes and gentle agitation on a roller table at room temperature until solubilised. Gentle mixing at 37 °C for 10 minutes can improve solubilisation of proteins.
11. Divide the solution into two equal aliquots and combine one with 800 μl of 1 M fresh hydroxylamine solution, 1 mM EDTA, protease inhibitors and 100 μl fresh 4 mM biotin-HPDP (Thermo scientific) dissolved in DMF and gently mix for 1 h at RT. Treat the remaining aliquot identically but replace hydroxylamine with 50 mM Tris pH 7.4.
12. Precipitate proteins at room temperature using methanol/chloroform as described previously.
13. Resuspend each sample in 100 µl of resuspension buffer and add 900 µl PBS containing 0.2% Triton X-100.
14. Remove 50 -100 µl to act as a loading control and chloroform/methanol precipitate as described previously and resuspend in 25 µl 2 x SDS-PAGE sample buffer.
15. Combine the remaining sample with 15 μl of high capacity neutravidin-agarose beads (Thermo scientific) in a microfuge tube for 1 hour at room temperature on a roller table.
16. Collect the neutravidin beads by centrifugation at 1,000 x g and wash with 1 ml wash buffer. Repeat.
17. Collect the beads and wash with 1 ml PBS.
18. Collect the beads and elute captured proteins in 25 µl of 2 × SDS sample elution buffer containing 40 % glycerol v/v and 1 % 2-mercaptoethanol v/v at 95 °C for 5 minutes.
19. Analyse samples by SDS/PAGE and Western blotting.

**General solutions**

**Lysis buffer**

1 x PBS pH 7.4

Protease inhibitors

1 mM EDTA

1 % Triton X-100

25 mM N-ethylmaleimide (Thermo scientific, prepare fresh)

**Resuspension buffer**

1 x PBS pH 7.4

8 M Urea

2 % SDS

**1M Hydroxylamine**

1 M Hydroxylamine in water (pH to 7.4 with NaOH)

**Wash buffer**

1 x PBS pH 7.4

NaCl to 500 mM

0.1 % SDS

**2× SDS sample elution buffer**

125 mM Tris pH 6.8

1 % β-mercaptoethanol

6 % SDS

0.005 % bromophenol blue w/v

40 % glycerol v/v
